# An accurate TMT-based approach to quantify and model lysine susceptibility to conjugation via N-hydroxysuccinimide esters in a monoclonal antibody

**DOI:** 10.1038/s41598-018-35924-0

**Published:** 2018-12-05

**Authors:** Jennifer J. Hill, Tammy-Lynn Tremblay, Christopher R. Corbeil, Enrico O. Purisima, Traian Sulea

**Affiliations:** 1 Human Health Therapeutics Research Centre, National Research Council Canada, 100 Sussex Dr, Ottawa, ON K1A 0R6 Canada; 2 Human Health Therapeutics Research Centre, National Research Council Canada, 6100 Royalmount Ave, Montreal, QC H4P 2R2 Canada

## Abstract

Conjugation of small molecules to proteins through N-hydroxysuccinimide (NHS) esters results in a random distribution of small molecules on lysine residues and the protein N-terminus. While mass spectrometry methods have improved characterization of these protein conjugates, it remains a challenge to quantify the occupancy at individual sites of conjugation. Here, we present a method using Tandem Mass Tags (TMT) that enabled the accurate and sensitive quantification of occupancy at individual conjugation sites in the NIST monoclonal antibody. At conjugation levels relevant to antibody drug conjugates in the clinic, site occupancy data was obtained for 37 individual sites, with average site occupancy data across 2 adjacent lysines obtained for an additional 12 sites. Thus, altogether, a measure of site occupancy was obtained for 98% of the available primary amines. We further showed that removal of the Fc-glycan on the NIST mAb increased conjugation at two specific sites in the heavy chain, demonstrating the utility of this method to identify changes in the susceptibility of individual sites to conjugation. This improved site occupancy data allowed calibration of a bi-parametric linear model for predicting the susceptibility of individual lysines to conjugation from 3D-structure based on their solvent exposures and ionization constants. Trained against the experimental data for lysines from the Fab fragment, the model provided accurate predictions of occupancies at lysine sites from the Fc region and the protein N-terminus (R^2^ = 0.76). This predictive model will enable improved engineering of antibodies for optimal labeling with fluorophores, toxins, or crosslinkers.

## Introduction

Conjugation of small molecules to antibodies and other proteins is widely used to produce both therapeutics and assay reagents^1^. Recently, antibody drug conjugates (ADCs) have been shown to be a promising approach for cancer therapy, combining the specificity of an antibody with the potency of small molecule toxins^2^. Several different conjugation chemistries have been utilized to attach molecules to reactive groups on amino acids, such as sulfhydryl groups on cysteine residues, or primary amines on lysine residues and the protein N-terminus. Alternative conjugation chemistries have also been described and are an area of intense development aimed to produce more stable and homogenous ADC products^3^.

Despite the high level of research on alternative conjugation schemes, many of the ADCs that are currently progressing to the clinic continue to be based on lysine conjugation of MCC-DM1^4^. MCC-DM1 consists of the cytotoxic agent emtansine (DM1) linked to the maleimide of the heterobifunctional crosslinker succinimidyl *trans*-4-(maleimidylmethyl)cyclohexane-1-carboxylate (SMCC)^4^. Conjugation of antibodies to MCC-DM1 proceeds through the reaction of the SMCC-derived N-hydroxysuccinimide (NHS) ester reactive group with primary amines on the protein N-termini and on lysine residues. Although standardization of the conjugation reaction conditions can produce ADCs with consistent average drug-antibody ratios (DARs) and drug distributions (percentage of IgG with a specific DAR), a broad population of species occurs since the NHS ester reacts with any accessible primary amine^5^. For example, mapping studies have found evidence of MCC-DM1 conjugation at more than 40 unique sites, on lysines or at the N-termini, in monoclonal antibodies^5,6^.

Generally, protein small-molecule conjugates are characterized on the basis of their DAR and drug distribution profile. An improved understanding of the susceptibility of individual lysine residues to conjugation may enable engineering efforts to minimize conjugation to sites that are required for biological activity. This knowledge would also improve our understanding of how changes to the antibody sequence, glycosylation profile, or other post-translational modifications may affect the final conjugated product. Site occupancy measurements would allow for a careful assessment of batch-to-batch variability under the controlled conditions chosen for conjugation. Finally, accurate profiling of conjugation susceptibilities would allow development of predictive structure-based molecular models that can potentially be used to bias conjugation towards a preferred subset of lysine residues in a controllable manner.

To date, however, it has proven difficult to accurately determine the relative level of conjugation at individual lysine residues, especially at the lower levels of conjugation typical for an ADC. For batch-to-batch comparisons, the MS signal intensity for each conjugated peptide was shown to effectively identify differences in site occupancy between samples^6^. However, this method did not provide information on the actual conjugation level at each site. Recently, several mass spectrometry (MS) approaches have been used to provide some information on site occupancy at individual lysines in ADCs by comparing the ion signal for conjugated peptides relative to unconjugated ones^7,8^. This can be done with or without correction for differences in ionization efficiency between these two species. However, these studies provided site occupancy data for a limited number of lysine sites, and the accuracy of their measurements was difficult to assess due to the inherent difficulties in comparing two different peptide species. These ‘apples-to-oranges’ comparisons are complicated by the fact that conjugation can affect not only the ionization and chromatographic properties of a peptide, but also the local susceptibility to protease digestion, even when non-specific or non-lysine dependent proteases are used.

One way to overcome some of the challenges associated with these ‘apples-to-oranges’ site occupancy measurements, would be to develop a workflow where both peptides (conjugated and unconjugated) are modified in a similar way. For example, in the context of protein crosslinking for structural elucidation, Jhan *et al*. have conjugated Cytochrome C to NHS-acetate to form a protein conjugate^9^. They then labeled all remaining lysines with a deuterium-containing acetate using anhydride chemistry. This method determined site occupancy values for most lysines in Cytochrome C. However, since the purpose of their work was to improve the results in protein crosslinking studies, they provided site occupancy measurements at relatively high levels of conjugation with minimal statistical analysis.

In this study, we have utilized tandem mass tags (TMTs) as a “toxin” surrogate in order to accurately quantify the susceptibility of individual lysine residues to conjugation, using a workflow that compares peptides of identical molecular structure. TMTs are conjugated to lysine and the protein N-terminus through the same NHS-based chemistry that is used to produce MCC-DM1 ADCs. TMTs have several advantages over toxins for this type of analysis, including their smaller size and most importantly, their well-defined fragmentation characteristics under MS-MS conditions which provide a unique mass tag for accurate quantification. Previous work by Gautier *et al*.^7^ has demonstrated the use of TMTs as surrogate ADCs to demonstrate the use of native mass spectrometry for intact mass analysis. In addition, they were able to approximate the relative reactivity of 43% of the available lysines, using a bottom-up approach that relied on quantification of the MS signal intensity of the conjugated peptide relative to the unconjugated one. However, this coverage dropped to 27% of the available lysines when labeling was done at levels commonly used in the production of ADCs, where only ~5 labels per antibody were added.

Here, we describe a novel workflow using TMTs as a surrogate ADC molecule that enables the accurate quantification and increased sensitivity of site occupancy at individual lysine residues with high coverage. We take advantage of the unique nature of TMTs by producing a surrogate ADC through the addition of TMT126 and then completely labeling all remaining primary amines in the intact, denatured protein with TMT127. In contrast to the TMT approach used by Gautier *et al*.^7^, this process enables direct determination of site occupancy for the TMT126 label by relying only on the ratio of the TMT126 label to the TMT127 label at any given site. By comparing peptides that are essentially identical in molecular composition and only differ in the position of a ^13^C isotope, this method prevents inaccuracies due to conjugation effects on protease susceptibility or ionization properties. Using this approach, we demonstrate that the site occupancy at individual lysine residues can be determined with a greatly improved level of accuracy, even at low conjugation ratios. This accurate profiling allowed us to develop a structure-based computational model of lysine susceptibility to conjugation, a predictive tool that can be useful for molecular engineering and optimization of future ADCs.

## Methods

### Production of TMT-conjugated antibody using TMT126

400 μg of NIST monoclonal antibody standard was diluted to 5 μg/μL and buffer exchanged into 1x conjugation buffer (0.1 M potassium phosphate, 20 mM NaCl, 2 mM EDTA, pH 7.2) using 2 subsequent Zeba Spin Desalting Columns (7 K MWCO, Thermo Scientific), as suggested by the manufacturer. The buffer exchanged NIST antibody was aliquoted into 3 tubes of 25 μl, containing 125 μg each. A vial of TMT126 reagent containing 0.8 mg, as provided by the manufacturer, was resuspended in 235 μl of acetonitrile (ACN). For the 30X molar excess condition, 2.5 uL (containing 8.5 ug TMT126) was added to the NIST mAb. Subsequently, the TMT solution was further diluted with ACN such that 2.5 ul contained the required amount of TMT126 for each remaining labeling condition (2.1 ug for 8X, 4.25 ug for 15X). All samples were incubated overnight at 25 °C in the dark. Following labeling, each antibody was diluted with 100 μL of PBS pH 7.4 prior to buffer exchange into PBS pH 7.4, as described above. For the deglycosylation experiment, 400 μg of NIST mAb was diluted to 5 mg/mL through the addition of 45 μL of PBS (control glycosylated sample) or PNGaseF (Sigma, deglycosylated sample) and incubated at 37 °C for 5 h, prior to the initial buffer exchange step.

### Intact mass analysis of TMT126-conjugated antibody

To remove sample heterogeneity due to glycans and C-terminal lysine residues prior to intact mass analysis, a 20 μg aliquot of each TMT-labeled sample was treated with 4 μL PNGaseF overnight at 37 °C followed by a 2 h incubation with 4 μL of Carboxypeptidase B (Sigma, 0.05 mg/mL). Samples were desalted using an Agilent HP1100 system with a 2.1 × 30 mm Poros-R2 reverse phase column (Applied Biosystems) by running a 3 min linear gradient from 20% to 90% Solvent B (Solvent A: 0.1% formic acid; Solvent B: 100% acetonitrile). Mass spectra were collected using an in line LTQ-Orbitrap XL (FTMS analyzer at 7500 resolution from 400–4000 Da). Spectra were summed over the protein elution range and the protein signal in the m/z range from 1800 to 3800 Da was deconvoluted using MaxEnt1. DAR values (in this work, the average number of attached TMT molecules per antibody molecule) were calculated using the weighted average of the MS signal intensity of each assigned TMT-conjugate species in the deconvoluted intact mass spectrum.

### Complete TMT-labeling of lysines with TMT127

Complete labeling was achieved following a modified version of the TAILS protocol^10^. Briefly, 100 μg of TMT126 labeled proteins were precipitated with 8 sample volumes of freezer cold (−20 °C) ACN and 1 sample volume of freezer cold methanol, incubated at −80 °C for at least 2 h and centrifuged. The protein pellet was washed twice with freezer cold methanol and briefly air dried. Each pellet was solubilized in 15 μL 6 M guanidine-HCl prior to addition of 25 μL dd-H_2_O and 10 μL 1 M HEPES, pH 8.0. Disulfide bonds were reduced and alkylated with 10 mM TCEP (30 min at RT) and 25 mM iodoacetamide (25 °C in the dark for 30 min). Each supplied vial of TMT-127, containing 0.8 mg of reagent, was resuspended in 110 μL of DMSO. For each sample, 55 μL of this TMT-127 solution and added to the reduced and alkylated protein, resulting in 50% v/v DMSO during labeling. Samples were incubated at 25 °C in the dark for 1 h, quenched with 100 μM ethanolamine, and incubated for an additional 30 min at RT to quench unreacted TMT labels. Unreacted reagents were removed by acetone/methanol precipitation as described above. Pellets were resuspended in 25 μL of 0.5% Rapigest in 50 mM ammonium bicarbonate (AMBIC) and diluted with 100 μL 50 mM AMBIC prior to buffer exchange into 50 mM AMBIC using a 7 K Zeba spin-column.

### Protease digestion

Each sample was diluted to 0.1 μg/uL with 50 mM AMBIC and split into three 25 μg aliquots for digestion with either (1) Trypsin (Promega, 1:20, 2–5 h at 37 °C)/GluC (Promega, 1:20, O/N at 37 °C), (2) Chymotrypsin (SIGMA C-3142, 1:40, O/N at 25 °C), or (3) Elastase (SIGMA E-0258, 1:20, O/N at 37 °C). Samples were stored at 4 °C or −80 °C prior to MS analysis.

### SDS-PAGE analysis

An equivalent of 1 μg of TMT-labeled NIST mAb after low labeling and high labeling were run on an SDS-PAGE gel (Bio-Rad). Proteins were visualized by Sypro ruby stain (Bio-Rad) as recommended by the manufacturer and imaged on a Biorad ChemiDoc MP Imaging system, using manufacturer suggested settings for Sypro ruby stained gels.

### Peptide-level LC-MS analysis

For the peptide-level analysis, 0.3 μg of digested protein was analyzed by automated nanoLC-MS(/MS) on an LTQ-Orbitrap XL (Thermo Scientific) coupled to a NanoAcquity UPLC system (Waters). Peptides were trapped using an inline C8 precolumn (LC Packings, 161194) and C18 trap column (Waters, 186003514) and separated on a 10 cm × 100 μm I.D. C18 column (Waters, 1.7 μm BEH130C18, 186003546) at ~250 nL/min using a 60 min gradient from 1% to 40% Solvent B (Solvent A: 0.1% formic acid, Solvent B: 100% ACN/0.1% formic acid), followed by a 3 min ramp to 85% Solvent B and a 9 min equilibration at 1% Solvent B. Blanks with a 25 min gradient were run between samples to minimize carryover. MS spectra were acquired in the Orbitrap between 400 and 2000 Da m/z in profile mode at 60k resolution, while data-dependent CID (ion trap, centroid mode) and HCD (Orbitrap, profile, 7500 resolution) scans of the top 2 ions were acquired with dynamic exclusion (20 s) using the following settings: isolation width = 2.0 HCD and 3.0 CID, activation Q = 0.250, activation time = 30 ms, and normalized collision energy = 45 HCD and 35 CID. All samples were injected a minimum of 2 times as technical repeats (TRs). In some runs, an inclusion list was used to provide quantification data on specific lysine containing peptides of interest. All samples were analyzed a minimum of 2 times for each digest enzyme.

### Database searches for peptide identifications

CID data was converted to mzXML using Msconvert from the ProteoWizard package^11^ with the following parameters: –mzXML -32 –filter ‘peakPicking true [2,3]’. MGF files (*.mgf) were generated from the mzXML file using MzXML2Search from the Trans Proteomics Pipeline project and searched with Mascot against a database containing the NIST mAb sequence with the following parameters: enzyme = none; modifications = carbamidomethyl (C, fixed), oxidation (M, variable), TMT-duplex (K and protein N-term, variable), Pyroglutamic acid (protein N-term Q); peptide tolerance = 1.2 Da; fragment tolerance = 1.2 Da. Peptides were subsequently filtered to remove peptides with a Mascot score <10 and a delta mass >5 ppm following correction for systemic mass error based the median ppm value of high scoring peptides (score >40). These filtering parameters lead to a false discovery rate (FDR) of <1%, based on a decoy search strategy against a scrambled NIST mAb sequence. As previously described^12^, we found the wide peptide mass tolerance followed by a tight mass filter led to a lower false identification rate than setting a tight mass tolerance during the search.

### TMT data analysis and statistics

The MS intensity of peaks with 20 ppm of the expected mass for the 126 and 127 reporter ions was extracted from the HCD MS/MS spectra and assigned to a Mascot search result based on the corresponding CID data. Site occupancy was calculated based on the intensity of the 126 reporter ion divided by the sum of the 126 and 127 reporter ion intensities. All spectra with a summed reporter ion intensity less than 120000 were removed from the analysis (see Results). Peptides that contained the same contingent of lysine residues were grouped and the average and standard deviation of the site occupancy values were calculated. Occupancy values were normalized based on experimental DAR values to allow direct comparison between technical replicates. All analyses were performed using in-house designed Perl scripts. Data from 2 to 3 individual experiments were combined in the final analysis.

The site occupancy of single lysine values calculated from peptides containing 2 or 3 lysines (‘doubles’ or ‘triples’ respectively) were determined using the following equations, where C is a correction value for the completeness of lysine labeling (C = 0.95 for data presented here):

1 $${\rm{Doubles}}:\,\,\,Occupanc{y}_{{\bf{Y}}}={\rm{C}}\,(2\cdot Occupanc{y}_{{\bf{X}}{\bf{Y}}}-Occupanc{y}_{{\bf{X}}})$$

2 $${\rm{Triples}}:\,\,\,Occupanc{y}_{{\bf{Y}}}={\rm{C}}\,(3\cdot Occupanc{y}_{{\bf{X}}{\bf{Y}}{\bf{Z}}}-2\cdot Occupanc{y}_{{\bf{X}}{\bf{Z}}})$$

### Calculation of molecular descriptors from 3D structures

The crystal structure of the NIST Fab (PDB code 5K8A) was used for the Fab fragment, with neutral capping (acetyl, ACE) of the heavy chain N-terminus and neutral capping (methylamino, NME) of the C-termini of both heavy chain (Cys223) and light chain (Cys213). The four independently refined Fab molecules present in the asymmetric unit were each prepared separately. The glycosylated Fc fragment including the hinge region (heavy chain D224-K450) was extracted from the crystal structure of a human IgG1 mAb (PDB code 1HZH), and neutral ACE groups were added at the N-termini of the dimeric Fc structure. The missing fragments T226-T228 in the hinge region of one heavy chain and P448-K450 from the C-terminus of the other heavy chain were reconstructed using the conformations observed in the complementary chain in Sybyl v8.1.1 (Tripos, Inc., St-Louis, MO) and then relaxed by constrained energy minimization with the AMBER force field^13,14^. The carbohydrate structures present in the crystal structure were retained and linked to N300 in the Fc H and K chains.

Molecular dynamics (MD) simulations were carried out with the AMBER16 CUDA software (University of California, San Francisco, CA) using the prepared crystal structures as input. The AMBER14SB^15^ and GLYCAM06j-1^16^ force fields were used to parameterize the protein and carbohydrates, respectively. A 12-Å truncated octahedron was used to solvate the system. Na and Cl counterions were added to neutralize the system to a final salt concentration of 0.1 M. TIP3P water parameters^17^ were used for the solvent. Each system first underwent an energy minimization where all heavy atoms were restrained using a harmonic potential with a force constant of 4 kcal mol^−1^ Å^−2^ followed by 30-ps NVT simulation to heat the system to 150 K, then 30-ps NPT simulation to heat to 300 K. This was followed by slowly removing the restraints on the side chain atoms of the protein over a 1-ns NPT simulation. A 10-ns production NPT run was obtained for each of the four Fab copies and for the Fc homodimer. Restraints having a harmonic potential with a force constant of 4 kcal mol^−1^ Å^−2^ were applied to the protein backbone and carbohydrate heavy atoms during the production run. To constrain the bond length of hydrogen atoms, we used SHAKE^18^. The time step was set to 2 fs and an 8-Å non-bonded cutoff was used. For long-range electrostatic treatment, particle mesh Ewald (PME)^19^ was used. Snapshots were taken every 100 ps over the last 8 ns of each simulation to generate conformational ensembles.

Prepared crystal structures (four Fab copies and two Fc chains) or generated MD conformational ensembles were then used to calculate average values for molecular properties. Acidity constants (p*K*_a_ = −log*K*_a_) of ionizable groups were calculated using the H++ ^20,21^, DelPhiPKa^22,23^ and PropKa^24,25^ programs. For pKa calculations with H++, recommended values for protein interior dielectric constant of 10, water dielectric constant of 80, and salt concentration of 0.15 M were used, whereas DelPhiPKa was run with default values of these parameters including a protein dielectric constant of 8. For both H++ and DelPhiPKa, the carbohydrate structures present in the glycosylated Fc fragment were parametrized with partial charges and atomic radii from the GLYCAM force field^16^. The PropKa empirical method was used with default parameters. Solvent-accessible surface area (SASA) calculations were carried out using a marching tetrahedral algorithm^26,27^ and scaled AMBER van der Waals radii^28^ increased by a water probe radius of 1.4 Å.

Multiple linear regressions (MLRs) were carried out with the glm function in R^29^. DAR-independent susceptibility values over experimental data at all three antibody:TMT126 ratios (1:8, 1:15 and 1:30) were used as the independent variable, and the calculated average molecular properties (p*K*_a_, SASA) as dependent variables. The assumptions of this model were that lysine reactivity would depend linearly on the surface exposure and the ionization potential of the side chain N atom, and that both properties would depend on the 3D structural environment. MLR models were trained on single lysine residues from the Fab fragment. Models were then externally tested on single lysine residues from the Fc fragment and the N-terminus of the Fab light chain, as well as on single lysine residues whose data was estimated from ‘doubles’ and ‘triples’.

## Results

### A two-step TMT labeling approach to enable accurate quantification of lysine site occupancy

**Figure 1:**
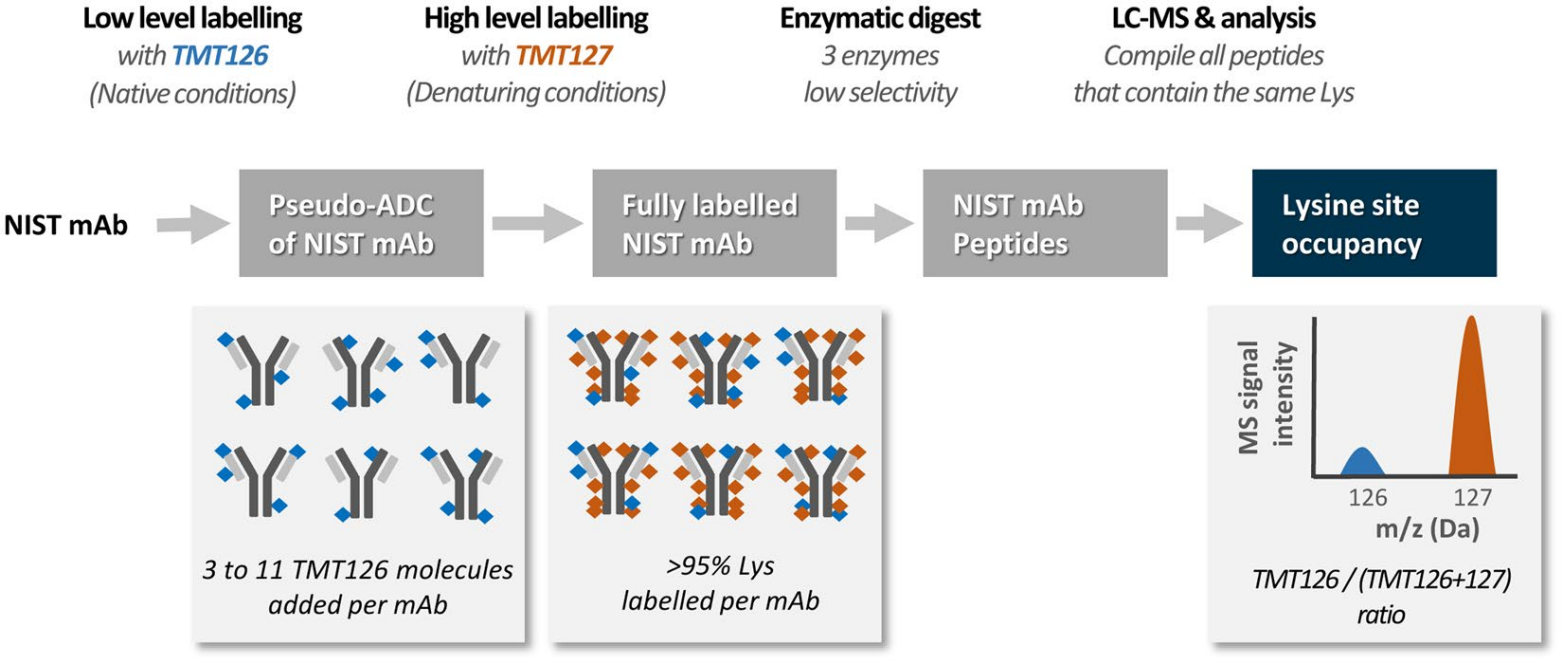
Concept and workflow. NIST mAb conjugates are produced by NHS ester-based conjugation of TMT126 under conditions which add an average of 3 to 11 TMT126 molecules per IgG molecule. The remaining available conjugation sites are then completely labeled with TMT127 under denaturing conditions. Following proteolytic digestion, the resulting peptides are subjected to LC-MS1 and MS2 analysis. An accurate quantification of the TMT126 site occupancy is obtained from the MS2 spectra derived from peptides containing a lysine or protein N-terminus, based on the ratio of the TMT126 reporter ion signal relative to the TMT127 reporter ion. TMT ratio data derived from all peptides that contain the same contingent of conjugated sites is combined to enable statistical analysis.

To determine the site occupancy of individual lysines after conjugation to an NHS-ester based molecule, we developed a method that relies on the relative quantitative accuracy enabled by the commercially available TMT reagents, which contain an NHS ester for conjugation. As depicted in Fig. 1, our proposed approach first uses the TMT126 reagent to produce pseudo-ADC conjugates of a monoclonal antibody (mAb). In these studies, we used the NIST mAb standard, a highly characterized member of the therapeutically-relevant IgG1k class of antibodies. Next, all remaining lysines in the antibody were conjugated to a complementary TMT, TMT127. Importantly, TMT127 has the same structure and molecular weight as the TMT126 used in the original conjugate and differs only in the location of a ^13^C isotope. This second-step labeling is performed under denaturing conditions with the aim of fully labeling all unconjugated lysines in the antibody. Finally, the fully labeled NIST mAb is digested with a variety of proteolytic enzymes to maximize the number of peptides available for analysis. The average site occupancy for the lysines present in a given peptide can be calculated by determining the ratio of the MS signal intensity of the TMT126 reporter ion to the total reporter ion intensity (TMT126 + TMT127).

**Figure 2:**
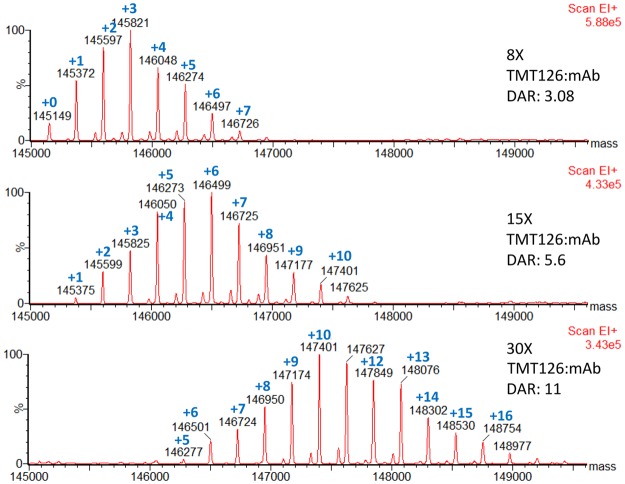
Mass spectrometry analysis of intact TMT126 conjugates to determine average number of TMT126 molecules added per antibody. Conjugates were produced by labeling under 3 different conjugation conditions differing only in the molar ratio of TMT label to antibody (8X, 15X, and 30X). To reduce heterogeneity, NIST-mAb was deglycosylated and the C-terminal lysine was removed with carboxypeptidase B prior to intact mass analysis. A peak corresponding to the expected mass of unconjugated intact NIST mAb (predicted: 145151 Da, observed “+0”: 145149 Da) was seen only in the 8X conjugation sample. For each conjugation condition, a series of peaks corresponding to the addition of ~225 Da are present, representing the addition of increasing numbers of TMT labels (+1,+2, etc). The average number of TMT molecules added to each NIST mAb molecule under each condition (DAR) was calculated based on the weighted average of the peak intensity for each population.

As proof-of-concept for this workflow, we conjugated NIST mAb to TMT126 under identical conjugation conditions using 8x, 15x, and 30x molar excess of TMT126 reagent to antibody. To characterize the resulting TMT126 conjugates, prior to the TMT127 labeling step, the intact conjugated antibodies were analyzed by mass spectrometry after addition of PNGaseF and carboxypeptidase B to reduce heterogeneity due to glycosylation or C-terminal lysine cleavage, respectively. Figure 2 shows the deconvoluted mass spectra from each of these conjugates. Each conjugate contains a distribution of molecules with different numbers of TMT moieties added. The average number of TMT126 molecules added to each antibody, commonly referred to as the drug-to-antibody ratio (“DAR”) in the ADC field, ranged from 3 to 11 for the different conjugation conditions that were used. As expected, increasing the amount of TMT126 in the conjugation reaction led to a linear increase in the number TMT labels, with an average of 3.0, 5.7, and 11.0 TMT molecules conjugated to each antibody for the 8X, 15X, and 30X conditions, respectively. Repeats of these conjugations under identical conditions proved to be quite consistent, with only minor variations in DAR (±0.2) and TMT-distribution profiles, even when the conjugations were performed months apart.

**Figure 3:**
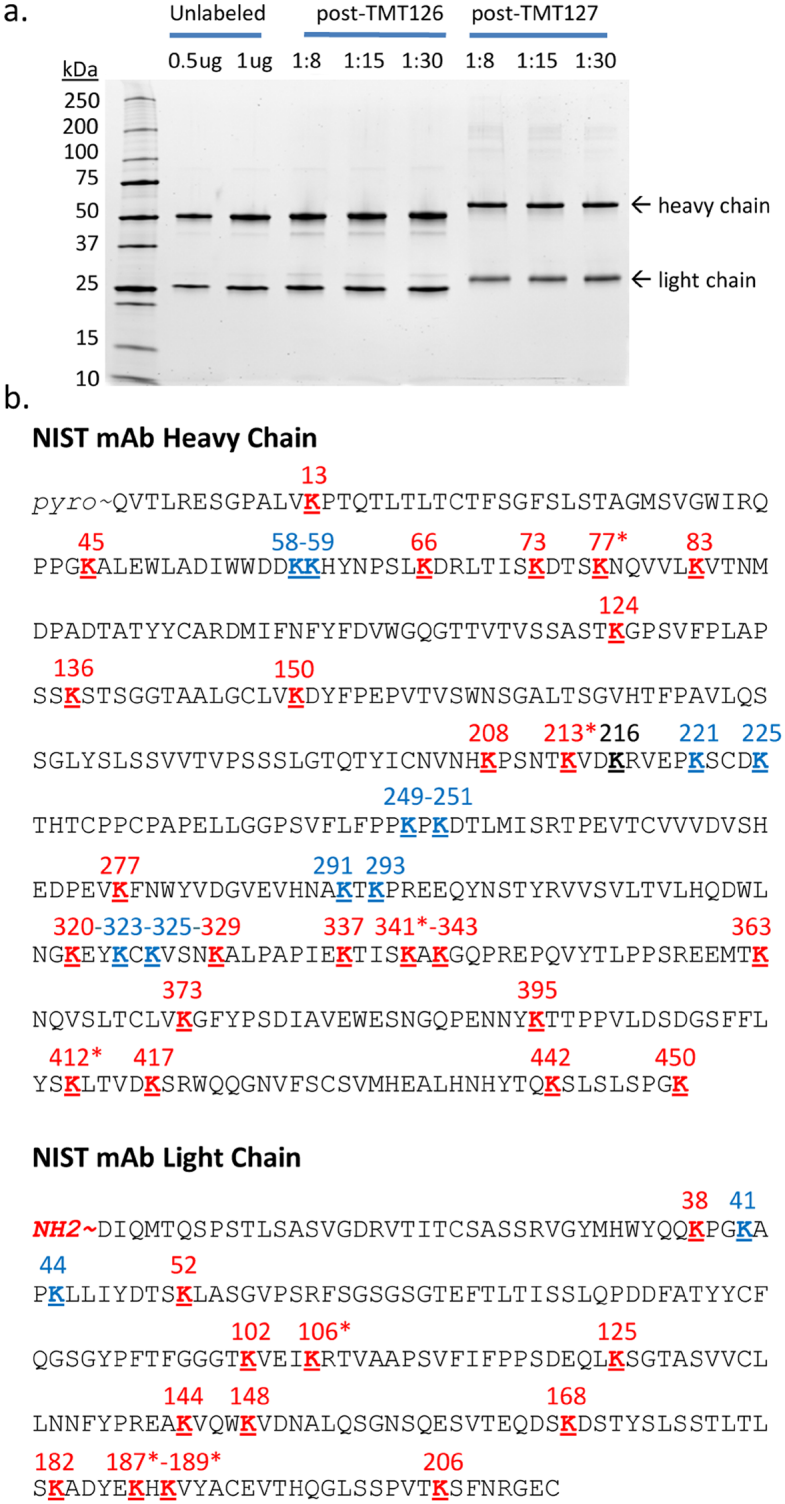
Accurate quantification of site occupancies at high sequence coverage. (**a**) NIST mAb was visualized by SDS-PAGE using a total protein stain, prior to conjugation with either 0.5 or 1 µg loaded on gel, after the TMT126 conjugation, and after the subsequent TMT127 conjugation steps. Although only minor mobility shifts are seen after the low-level TMT126 labeling, the TMT127 labeling results in a strong upward shift in the apparent molecular weight, indicative of successful labeling. The entire SDS-PAGE separating gel is shown. (**b**) Proteolytic digestion with Trypsin/GluC, Chymotrypsin, and Elastase enabled a high coverage of lysines and protein N-termini for site occupancy determinations, as shown on the amino acid sequence of the heavy chain and light chain of the NIST mAb. Individual site occupancy data was found for all conjugation sites numbered in red. Most of these were obtained from peptides which contained only a single site of conjugation. However, sites marked with an asterisk (*) were calculated using occupancy values obtained from peptides containing two or three lysine residues. In addition, we were able to determine average site occupancy data for 2 adjacent lysines for an additional 12 sites, labeled in blue, providing a measure of site susceptibility for 49 of the possible 50 sites. Only a single site (HC-216, labeled in black) did not have any site occupancy data in this analysis.

Accurate quantification with our proposed workflow requires high efficiency in the second step TMT127 labeling reaction such that nearly all primary amines in the monoclonal antibody (lysine residues and protein N-termini) are labeled. To evaluate this, we subjected the NIST mAb TMT126 conjugates to our full TMT127 labeling protocol and then estimated the efficiency of complete lysine labeling in two ways. First, we visualized the proteins on an SDS-PAGE gel after the first TMT126 labeling and after the second TMT127 labeling. As can be seen in Fig. 3a, there is a clear upward shift in the apparent molecular weight of both the heavy chain and light chain following the TMT127 labeling and the post-TMT127 sample appears as a single tight band in this analysis. As a second approach, we used Mascot to search the MS/MS data collected from LC-MS(/MS) analysis of peptide digests of the post-TMT127 labeled samples. In the search parameters, we included the TMT-label mass as a variable modification on lysine and then compiled all lysine-containing peptides with a unique m/z. This approach led to the identification of 638 peptides where all lysines were modified with a TMT label and only 21 peptides that contained an unlabeled lysine (3%).

Most ADCs are conjugated to an average of three or four toxins per molecule, suggesting that the site occupancy at any given lysine is likely to be quite low, especially considering that antibodies typically have more than 80 lysine residues available for conjugation. In order to determine if our method would have the necessary dynamic range to analyze antibody conjugates at these low levels, we examined the 126 and 127 reporter ions produced by individual peptides from the 8x NIST mAb conjugate. Evaluation of this data revealed that many spectra with a quantified TMT127 reporter ion did not have a detectable signal for the TMT126 reporter ion. While it is possible that this reflects a low level of TMT126 conjugation at this lysine, it is more likely to reflect a sensitivity limit in our mass spectrometry analysis. Based on this observation, we decided to empirically determine the minimum intensity value of the TMT127 reporter ion required to reliably detect the TMT126 reporter ion signal, if present. To evaluate this, we used the TMT data collected from this 8x NIST mAb conjugate to calculate the positive likelihood ratio of TMT126 detection using different cutoff values for the minimum intensity of the TMT127 reporter ion (Supplementary Figure S1). In this context, the positive likelihood ratio acts as a measure of the “detectability” of the TMT126 reporter ion signal at each TMT127 intensity value, while also considering the loss of data points due to overly stringent filtering. As seen in Supplementary Figure S1, the maximum positive likelihood ratio is reached at a reporter ion intensity of 120000. Thus, in all future experiments, we only include data from peptides with a reporter ion intensity above this threshold.

### Lysine susceptibility to NHS-based conjugation in NIST mAb

To determine the site occupancy at individual lysine residues, the peptide digests from NIST mAb samples conjugated at the three different ratios (8x, 15x, 30x) were analyzed by MS and the TMT ratios, representing site occupancy of the TMT126 label, were calculated for all spectra. Subsequently, all peptides containing the same cohort of lysines were grouped and their average TMT ratio was calculated. As can be seen in the individual peptide data shown in Supplementary Dataset S1, all peptides that contained the same cohort of lysines had very similar site occupancy values to each other, even when the parent peptides had differing amino acid compositions overall. Site occupancy data was obtained for most of the lysines present in the NIST mAb, as depicted in Fig. 3b, using site occupancy values measured for 29 single lysines and the light chain N-terminus, in addition to 16 peptides that contained 2 different lysines and 8 peptides that contained 3 lysines. The TMT ratios of these ‘double’ and ‘triple’ peptides represent an average occupancy of all sites that are present in the peptide. In some cases, it was possible to mathematically determine individual site occupancy values based on different combination of peptides (see Methods). This analysis provided data for an additional 7 single lysines, marked with an asterisk in Fig. 3b. In this way, site occupancy data was collected for 37 individual conjugation sites, representing 74% of the sites available for amine-based conjugation in the NIST mAb. An additional 12 sites are found exclusively in ‘double’ or ‘triple’ peptides without sufficient data available to determine site occupancy at each individual site; however, the average occupancy across the two or three sites was determined. When including these average occupancy values, our analysis was able to provide an indication of site occupancy for 49 of the possible 50 sites, representing coverage of 98% of available primary amines. Altogether, this dataset provided information on lysine susceptibility to conjugation with NHS-esters for all except one lysine (K216 in the HC) in the NIST mAb sequence.

**Figure 4:**
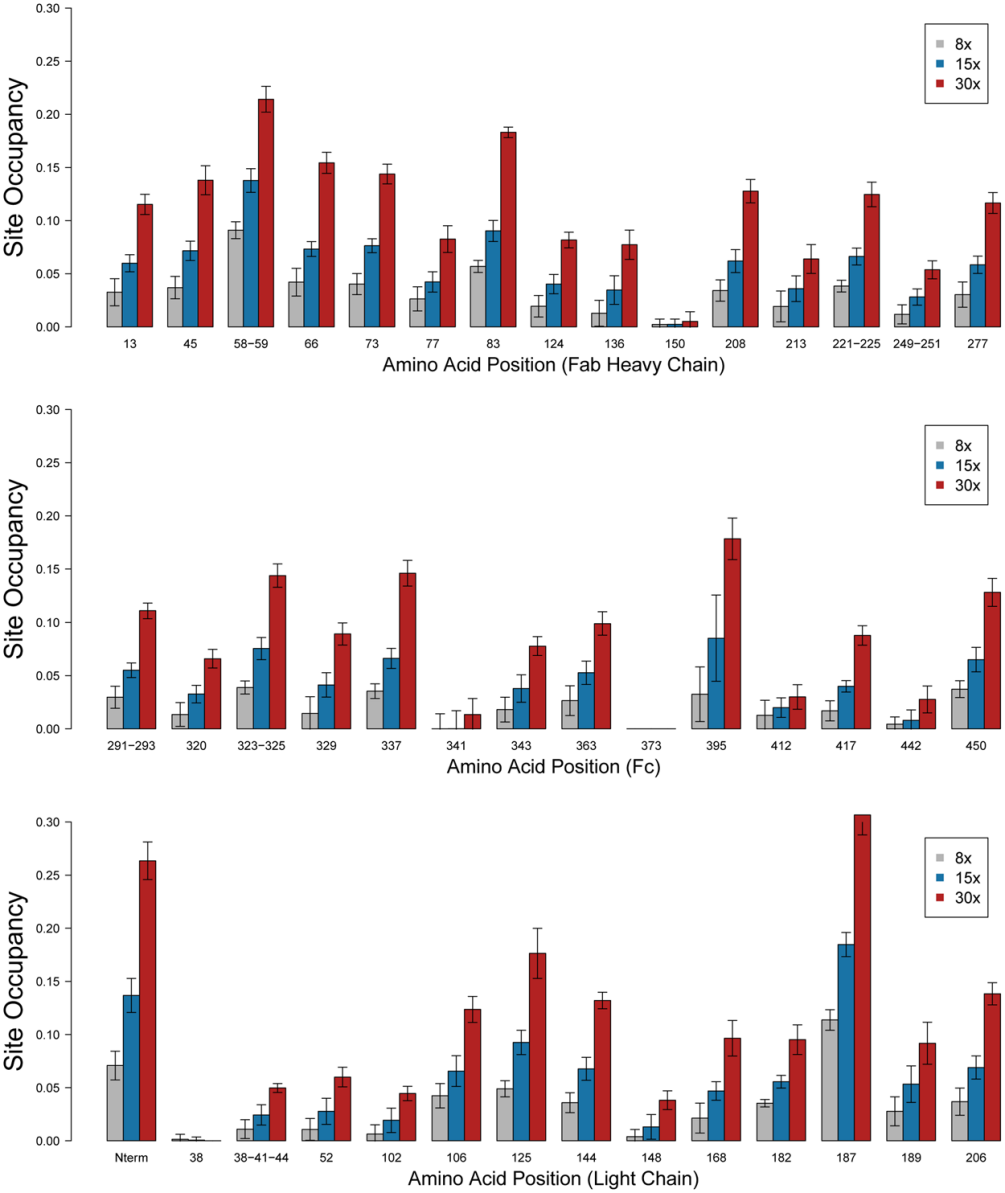
Site occupancy data for the 8X, 15X, and 30X TMT126 conjugates. The average site occupancy ratio is plotted for each lysine and for the protein N-termini of the NIST mAb standard, under each of the three conjugation conditions tested. As demonstrated by the error bars, representing the 95% confidence interval, the data showed a high-level of consistency between experiments.

The final site occupancy data from this complete analysis are depicted in Fig. 4 with detailed numerical data and statistics provided in Supplementary Dataset S2. As expected, the site occupancy for the conjugates with a higher DAR, as determined by our intact mass analysis in Fig. 2, is reflected in a higher site occupancy at each individual lysine. The average site occupancy per lysine was 2.9%, 5.9%, and 10.5% for the 1:8, 1:15, and 1:30 conjugates respectively. Individual lysines, however, displayed a range of site occupancy values. For example, some amines, such as K58/K59 in the heavy chain (HC), the K187 in the light chain (LC), and the N-terminus of the LC, are very amenable to conjugation, as reflected in their relatively high site occupancies. In contrast, other lysines, including K150, K341, K373 in the HC and K38 in the LC, show a very low level of conjugation, suggesting NHS attachment at these sites is unfavorable. As reflected by the error bars representing the 95% confidence interval, the quantification provided by this method is quite accurate. The accuracy of this method is improved with higher site occupancy values which better absorb minor inaccuracies in the quantification of the TMT reporter ion intensities, as reflected by the average relative standard deviation (RSD) for all measured site occupancy values, which ranges from 60% in the low level conjugate (1:8 sample) to 17% in the higher level conjugate (1:30 ratio) (Supplementary Figure S2).

**Figure 5:**
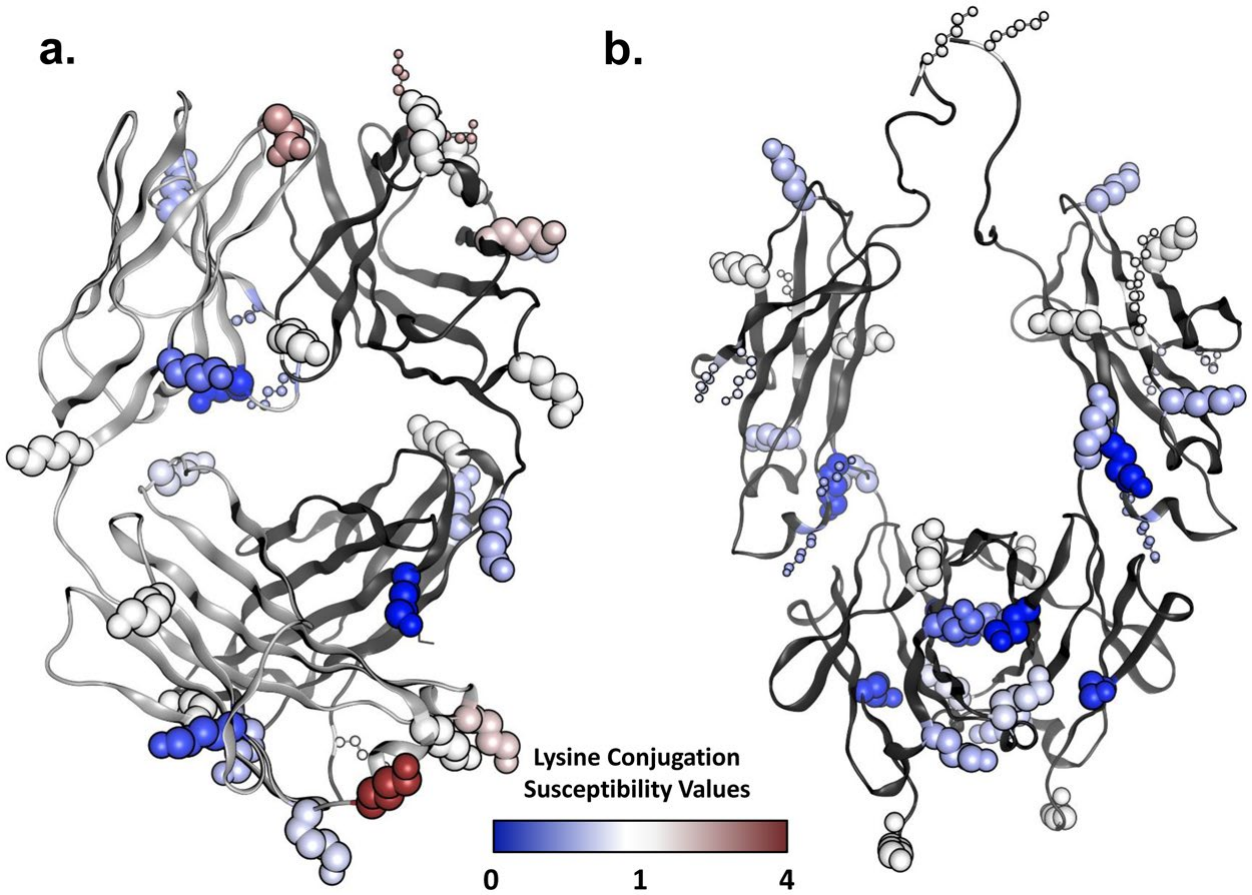
Mapping of lysine and protein N-terminus susceptibilities on the 3D structure. (**a**) Fab domain of NIST antibody; (**b**) Fc domain of NIST antibody. Lysine side chains are shown as CPK models, except for lysines with data estimated from ‘doubles’ and ‘triples’, which are shown as ball-and-stick models. Each lysine side chain is then color coded according to the susceptibility data from Table 1 as per the color scale legend shown.

In general, it appears that most if not all sites respond in a linear manner to an increase in TMT label present during conjugation, suggesting that each lysine has a particular conjugation probability that does not depend on DAR. We have taken advantage of this observation to calculate a conjugation susceptibility factor that is independent of the DAR, by normalizing the observed site occupancy values to the expected average occupancy value if all available conjugation sites were conjugated equally. By doing this, a conjugation susceptibility factor of 1 represents a site that is conjugated with a site occupancy equal to the expected site occupancy value based on the DAR alone. The conjugation susceptibility factors for the NIST mAb is shown in Table 1 and mapped onto the crystal structures of the NIST Fab and Fc fragments in Fig. 5.

**Table 1:** Susceptibility of lysines in the NIST mAb to NHS-ester conjugation. Conjugation Susceptibility Values: 1 indicates expected level of conjugation based on DAR if all conjugation sites were equal.

	Lysine AA Position	Conjugation Susceptibility Value	Standard Deviation	N
NIST mAb heavy chain	13	1.06	0.25	133
	45	1.25	0.22	276
	58–59	2.47	0.20	89
	66	1.35	0.25	71
	73	1.33	0.21	93
	77*	0.79	0.25	93
	83	1.69	0.17	17
	124	0.70	0.21	104
	136	0.58	0.27	66
	150	0.05	0.12	39
	208	1.13	0.26	53
	213*	0.62	0.34	46
	221–225	1.19	0.15	84
	249–251	0.46	0.19	219
	277	1.03	0.24	130
	291–293	0.98	0.19	55
	320	0.54	0.23	145
	323–325	1.31	0.17	53
	329	0.68	0.30	55
	337	1.20	0.18	19
	341*	−0.05	0.36	39
	343	0.66	0.26	160
	363	0.91	0.28	129
	373	0.00	0.00	29
	395	1.37	0.70	32
	412*	0.37	0.35	38
	417	0.66	0.23	38
	442	0.17	0.17	222
	450	1.18	0.21	24
NIST mAb light chain	Nterm	2.39	0.30	26
	38	0.02	0.09	72
	38-41-44	0.41	0.21	66
	52	0.48	0.22	158
	102	0.32	0.21	174
	106*	1.23	0.27	164
	125	1.62	0.22	61
	144	1.19	0.23	49
	148	0.24	0.18	163
	168	0.81	0.25	51
	182	1.00	0.12	32
	187*	3.27	0.24	32
	189*	0.90	0.32	105
	206	1.23	0.26	254

Asterisks mark calculated values.

### Effect of Fc-glycosylation on lysine susceptibility to conjugation with NHS esters

**Figure 6:**
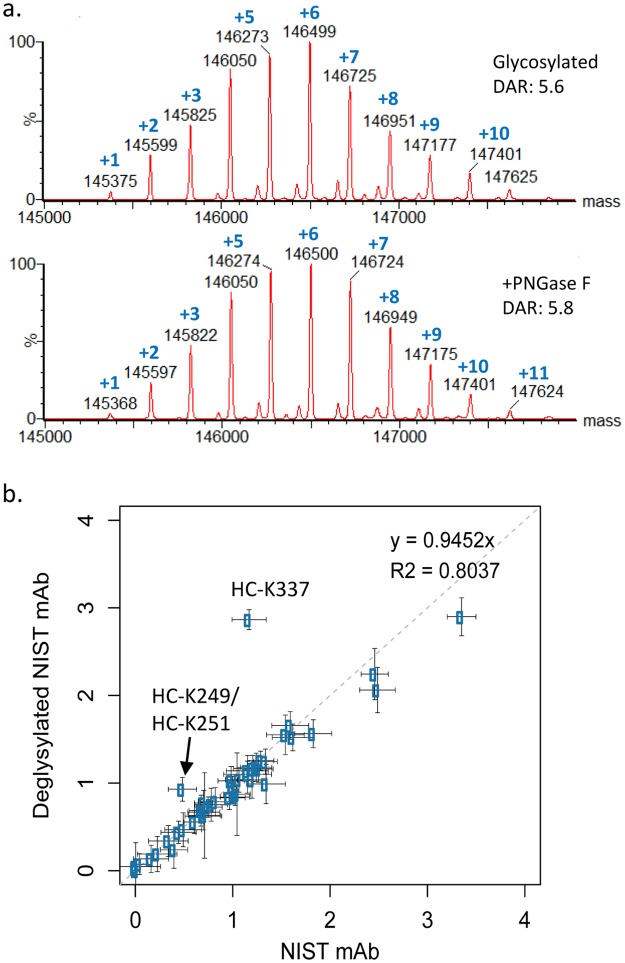
The Fc glycan affects the susceptibility of 2 heavy chain lysines to conjugation by NHS esters. (**a**) Intact mass analysis shows that conjugation of NIST mAb with TMT126 after removal of the Fc glycan with PNGaseF (‘+PNGaseF’) results in a similar DAR and distribution pattern to NIST mAb with the Fc glycan remaining (‘Glycosylated’). (**b**) Comparison of lysine susceptibility values between standard NIST mAb and deglycosylated NIST mAb identifies one single lysine (heavy chain K337) and one double lysine pair (HC-K249/HC-K251) that demonstrate a difference in conjugation susceptibility due to removal of the Fc-glycan with PNGaseF prior to conjugation, suggesting that the Fc glycan plays a role in the susceptibility of these lysines to conjugation via NHS-esters. The dotted line indicates perfect correlation.

Next, we utilized our method to determine whether the presence of a glycan at the N-linked site in the Fc region of the NIST antibody affects the susceptibility of individual lysines to conjugation with NHS esters. To do this, we produced 15x conjugates of deglycosylated NIST mAb. Prior to conjugation, NIST mAb was deglycosylated by treatment with PNGaseF, an enzyme that removes the N-linked glycan normally present at position 300 in the Fc region of the heavy chain, replacing it with an aspartic acid and the site susceptibility values were compared to those found in the glycosylated NIST mAb. As shown in Fig. 6, the susceptibility of most lysines to conjugation was not affected by deglycosylation of the NIST antibody. However, significant increases in conjugation were noted at NIST mAb peptides containing K249/K251 and K337 in the heavy chain in the deglycosylated sample. The 3D crystal structure suggests that these glycosylation effects on the site occupancy at heavy chain positions K249 and K337 labeling can be explained by interactions of carbohydrates with these lysines, which limit their accessibility in the glycosylated state (Supplementary Figure S3).

### A model to predict lysine susceptibility based on 3D structure

It is expected that conjugation susceptibilities of various residues relate to the reactivity of their primary amine, which in turn should depend on solvent exposure and nucleophilicity. Both parameters are 3D-structure dependent and are affected by the local geometry and electrostatics around each lysine residue. Hence, a bi-parametric linear model of lysine conjugation susceptibility was derived by describing solvent exposure by the solvent-accessible surface area (SASA_N_) and nucleophilicity by the acidity constant (p*K*_a_) at aliphatic N atoms of the NIST mAb. Model calibration was done on the set of 18 Lys residues from the Fab fragment with measurements based on peptides containing a single TMT-labeled lysine and averaged parameters calculated from the 4 crystal copies of the NIST mAb. This led to the following linear regression equation:

3 $$Susceptibility=0.0091\cdot {{\rm{SASA}}}_{{\rm{N}}}-0.6397\cdot {\rm{p}}{{K}}_{{\rm{a}}}+7.5824$$

**Figure 7:**
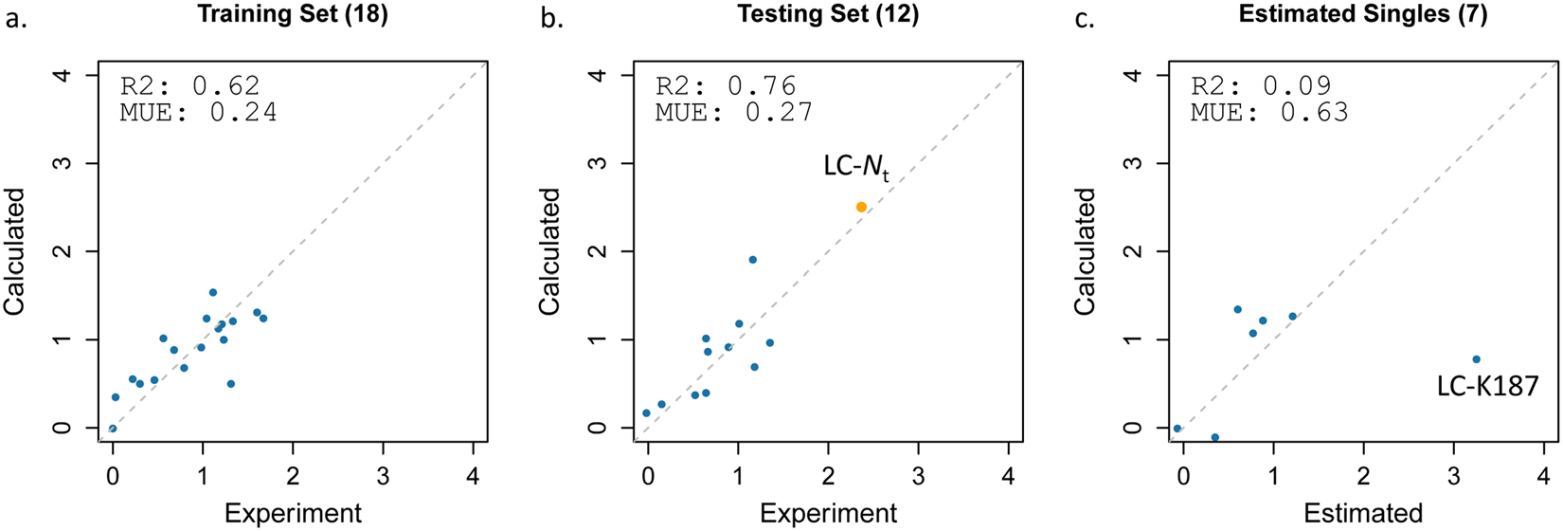
Performance of the bi-parametric linear model of amine susceptibilities to conjugation via NHS esters. Experimental DAR-independent susceptibility values are from Table 1. Only sites corresponding to peptides with a single lysine are used. Calculated molecular descriptors are averages over crystal structure conformations of the Fab and Fc fragments (see Methods). (**a**) Model training on 18 lysines from the Fab fragment. The model corresponds to Eq. (3). (**b**) Model testing on 11 lysines from the Fc fragment plus the N-terminus of the light chain (orange symbol). (**c**) Model testing on 7 lysines with susceptibility values estimated from data for ‘doubles’ and/or ‘triples’.

This fitted linear model achieves a good correlation with R^2^ = 0.62 (Fig. 7a) and a mean-unsigned-error (MUE) of 0.24 units on the DAR-independent susceptibility scale. The model indicates increased susceptibility to conjugation with increased surface accessibility (positive SASA_N_ coefficient) and with decreased probability of protonation (negative p*K*_a_ coefficient) at the N atom, in line with the expected physics for the conjugation reaction. As shown in Table 2, the p*K*_a_ descriptor makes a larger contribution to this model (R^2^ = 0.56; MUE = 0.29) than the SASA_N_ descriptor (R^2^ = 0.19; MUE = 0.38).

**Table 2:** Performance of various structure-based linear models of conjugation susceptibility.

Model Descriptors	R^2^		MUE^b^	
	Training set Fab(N = 18)	Testing set Fc(N = 12) + Nt	Testing set Fab(N = 18)	Training set Fc(N = 12) + Nt
Null^a^	0.00	0.00	0.44	0.45
SASA_N_	0.19	0.05	0.38	0.42
pKa(H++)	0.56	0.78	0.29	0.29
pKa(DelPhiPKa)	0.49	0.75	0.32	0.32
pKa(PropKa)	0.01	0.48	0.43	0.41
SASA_N_; pKa(H++)	0.62	0.76	0.24	0.27
SASA_N_; pKa(DelPhiPKa)	0.50	0.76	0.31	0.30
SASA_N_; pKa(PropKa)	0.40	0.78	0.32	0.26

^a^Null model is based on average experimental value for the given set.

^b^Mean-unsigned-error (MUE) in DAR-independent susceptibility units.

In order to further test its robustness, the bi-parametric model was employed for external predictions of conjugation susceptibilities at the 11 Lys residues from the Fc fragment, for which data could be obtained from peptides with a single TMT-label. Also included in this external prediction was the conjugation susceptibility at the N-terminus of the LC. Parameters were calculated based on a crystal structure of the Fc homodimer and averaged between the two chains for each lysine site. As seen in Fig. 7b showing predicted versus measured susceptibilities, the linear model in Eq. (3) exhibits significant predictive power with a high level of correlation to experimental data (R^2^ = 0.76; MUE = 0.27; see also Table 2). Finally, the model was tested against the set of 7 lysine residues with susceptibility estimated from measurements based on peptides with 2 and/or 3 TMT-labeled lysines (i.e., ‘doubles’ and ‘triples’). While significant statistics cannot be extracted due to the small size of this subset, it is visually apparent from Fig. 7c that the model makes reasonable predictions at 6 lysine sites, with the one noteworthy outlier being LC-K187 for which the SASA/p*K*_a_ model severely underestimates its susceptibility to conjugation.

The model in Eq. (3) was based on p*K*_a_ values calculated with the H++ method^20,21^. Since computational p*K*_a_ predictions are considered to be relatively challenging, we have attempted to test other alternative methods. We employed DelPhiPKa^22,23^, which like H++ includes a relatively high-level treatment of solvation effects, both using Poisson-Boltzmann electrostatics. DelPhiPka lead to models that compare favorably with those based on H++, albeit with a weaker performance (Table 2). We also tested the empirical method PropKa^24,25^, which however led a more marked decrease in performance.

Finally, we tested the impact that structural flexibility has on the quality of the susceptibility model in Eq. (3), which was derived based on only a few crystallographic conformations of the protein. We generated MD conformational ensembles starting from each crystal conformation and recalculated the SASA and p*K*_a_ values as averages over conformational snapshots along the MD trajectories. As shown in Supplementary Figure S4 for the Fab domain, using MD ensembles seems to reduce the variability in model quality as compared with the corresponding individual crystal structures. However, over all MD trajectories the model was not improved relative to the model averaged over the few crystal structures (Supplementary Figure S5). All calculated molecular properties based on crystal structures and MD ensembles are provided in Supplementary Dataset S3.

## Discussion

In this study, we describe a novel method using TMTs to directly measure the site occupancy at individual sites on a monoclonal antibody following conjugation via an NHS-ester reactive group. The TMT molecule offers several advantages in the described approach, most importantly, the very high level of similarity between the TMT126 and the TMT127 molecules, which differ only in the position of a 13 C isotope label. Our method relies on the use of one TMT (TMT126) to make the conjugate and subsequent labeling of the same sample with the other TMT (TMT127) under denaturing conditions such that TMT-127 is added to all remaining lysines, with an efficiency of approximately 95%. Using this approach, the ratio of the TMT126 signal to the TMT127 signal can be used directly to calculate site occupancy. This approach avoids comparing a modified to an unmodified peptide, eliminating inaccuracies due to differences in ionization properties or protease susceptibility due to the presence of the modification.

TMTs have been previously used to estimate the site occupancy of NHS-conjugation in a monoclonal antibody by Gautier *et al*.^7^. However, the approach that they described is fundamentally different from the one described in this work, as depicted in Supplementary Figure S6A. Gautier *et al*. used TMTs to label a monoclonal antibody under different conditions such that each TMT reporter ion represented an individual antibody conjugate sample with various numbers of TMT molecules attached. Thus, in their approach, the TMT reporter ions were used to determine the *relative* level of TMT addition between different samples. Gautier *et al*. then relied on a comparison between the MS signal intensity of each TMT-conjugated peptide to its equivalent unmodified peptide, in a separate MS run, to estimate the actual site occupancy values. There are 35 lysines in common between the NIST mAb used in our study and the IgG1 used by Gautier *et al*. Comparing the 120 μM labeling condition in Gautier *et al*. with our 30X labeling condition (DAR = 13 vs 11, respectively), Gautier *et al*. report measurable occupancy values (above 0) for 12 of the 35 sites that are in common between our 2 antibodies. Here, we describe measurable site occupancy values for 26 of these individual sites, a 2-fold improvement in coverage. Furthermore, this work provides average occupancy data between 2 adjacent lysines for an additional 8 of these sites found in ‘double’ peptides. For many sites, the occupancy values that we have obtained show similar trends to those obtained by Gautier *et al*., with a few exceptions (Supplementary Figure S6B). However, the majority of the occupancy values obtained by Gautier *et al*. were considerably higher than the ones obtained here, possibly reflecting a difference in the MS signal intensity of TMT conjugated peptides over unconjugated ones.

The NIST mAb has 35 unique lysines in the heavy chain and 14 unique lysines in the light chain plus one available N-terminus on the light chain. We did not see conjugation at the heavy-chain N-terminus as the NIST mAb contains a pyroglutamic acid at this site. Since an antibody molecule contains two identical heavy chains and two identical light chains, a total of 100 primary amines are available for conjugation in the intact IgG molecule. In the study described here, we produced a series of conjugates with increasing DAR values, as measured by intact mass analysis. Although our results show that individual lysine residues show variability in their susceptibility to conjugation by NHS esters, the average site occupancy for molecules in this DAR series, as calculated based on our method, averaged 2.9%, 5.9%, and 10.5% for molecules with DAR of 3.0, 5.7, and 11.0 respectively – very close to the predicted values of 3.0%, 5.7%, and 11.0% calculated from the DAR values. These findings are reassuringly close to the expected values. Furthermore, by summing the site occupancy values for all measured sites, the results agree well with the DAR values identified by intact mass: 2.8, 5.3, and 10.3. The site occupancy values are also quite consistent among replicates conjugated at different times, suggesting that the site occupancy at a given lysine is a feature of the protein and not simply the results of a stochastic process. This consistency enabled us to develop a lysine susceptibility value that is independent of the total level of conjugation and DAR.

In this analysis, several peptides contain multiple lysine residues, such that the ratio of TMT126-127 corresponds to an average of the site occupancy at each individual lysine. In some instances, it proved possible to calculate the individual site occupancies from data collected for different overlapping lysine groupings. However, in others, this was not possible as the lysines were not effectively separated by any of the 3 proteolytic enzymes used. It may be possible to separate these lysines using alternative enzymes, such as pepsin, at the expense of adding to the analysis time. Alternatively, it may be possible to use MS3 data to collect TMT ratios for individual peptides through fragmentation of MS2 ions that only contain a single lysine. While we were unable to achieve sufficient sensitivity with our mass spectrometer for this analysis, newer instruments may facilitate this approach, improving coverage for individual lysines. Building an effective MS3-based workflow for this analysis may also lead to significant savings in analysis time. Theoretically, if MS3 can directly identify individual site occupancy values for each of the 2 or 3 sites present in a ‘double’ or ‘triple’ peptide, a single protease digestion may be able to provide similar coverage levels to the 3 different digestion conditions that were used in this work.

The recording of lysine conjugation susceptibilities at high resolution and coverage provided by this study allowed the development for the first time of a robust structure-based computational model of susceptibility. This simple 3D-structure based linear model highlights the nitrogen atom’s surface exposure (SASA) and nucleophilicity (described by p*K*_a_) as the main factors to lysine reactivity. Importantly, the present model quantitates the key contribution of nucleophilicity to predicting the reactivity of surface-exposed lysines. To that end, the computational treatment of solvation effects for p*K*_a_ calculations was found to be critical to the development of a high-correlation model. It is also noteworthy that averaging descriptors over multiple conformations, either taken from crystal structures or simulated MD ensembles, was also important to obtaining correlative models. The contributions of nucleophilicity and structural flexibility to conjugation were not evaluated quantitatively in the previous TMT-based study^7^, which also afforded a more limited coverage unsuitable for statistical model training and testing as it was carried out in the present study. An intriguing question is whether there are other meaningful structure-based molecular descriptors that can be considered in order to further account for the variance in the conjugation susceptibility data. The case in point is K187 in the light chain of NIST mAb (constant region of the human kappa chain) which is underestimated by the present SASA/p*K*_a_ model.

The robustness of the calibrated SASA/p*K*_a_ model in external testing suggests that it can also be employed in the predictive mode. An immediate useful application will be evaluating the risk that lysines present in the antigen-binding regions (CDR) of antibodies will affect biological activity by estimating their likely susceptibility levels. The model can also be generally applied for predictions of amine susceptibility to conjugation via NHS esters for any protein as long as 3D-structural data are available or a homology model can be built. In this regard, we found that the worst model based on an MD ensemble was superior to the worst model based on a single crystal structure (Supplementary Figure S4). This provides a strategy to performing predictions with the proposed SASA/p*K*_a_ model on antibodies and proteins for which multiple crystal structure will not be available.

The same TMT based approach could also be used to analyze conjugation reactions beyond NHS ester chemistry. Commercial TMT tags are sold for cysteine conjugation via alkylation with an iodo group. With some effort to optimize the complete labeling step, additional conjugation chemistries could be explored through this method by synthesizing additional molecules with TMT-like properties. It is likely that the structure-based computational model will need to be retrained and tested for the specific conjugation reagent and reactive residues of the protein. In conclusion, the TMT method described here, combined with molecular modeling, will enable improved engineering of antibodies for optimal labeling with fluorophores, toxins, or crosslinkers and will provide the basis for the development of proteins with improved conjugation properties.

## Electronic supplementary material

Supplementary Information

Supplementary Dataset S1

Supplementary Dataset S2

Supplementary Dataset S3
