# Supplementary material for: An accurate TMT-based approach to quantify and model lysine susceptibility to conjugation via N-hydroxysuccinimide esters in a monoclonal antibody
Source: Sci Rep. 2018 Dec 5;8:17680. doi: 10.1038/s41598-018-35924-0 (PMC6281681; doi:10.1038/s41598-018-35924-0)
Supplement: Supplementary file 1 — Supplementary Information [file 41598_2018_35924_MOESM1_ESM.pdf]

Supplementary Material

for

An accurate TMT-based approach to quantify and model lysine susceptibility to conjugation via N-hydroxysuccinimide esters in a monoclonal antibody

Jennifer J. Hill[1,*], Tammy-Lynn Tremblay[1], Christopher R. Corbeil[2], Enrico O. Purisima[2] and Traian Sulea[2,*]

Human Health Therapeutics Research Centre, National Research Council Canada, [1] 100 Sussex Dr., Ottawa, ON, K1A 0R6, Canada, and [2] 6100 Royalmount Ave., Montreal, QC, H4P 2R2, Canada

*Corresponding authors: jennifer.hill@nrc-cnrc.gc.ca, 613-993-7206; traian.sulea@nrc-cnrc.gc.ca, 514-496-1924

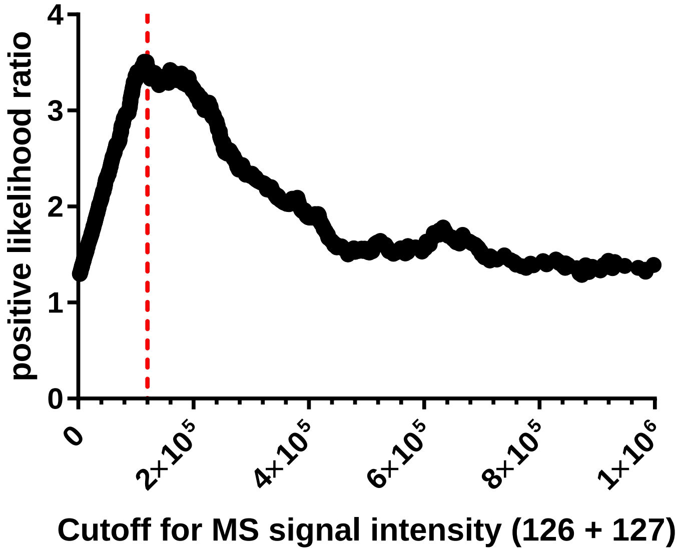

Supplementary Figure S1. Empirical determination of minimum TMT signal intensities required for accurate TMT quantification in an 8X conjugated NIST mAb sample.

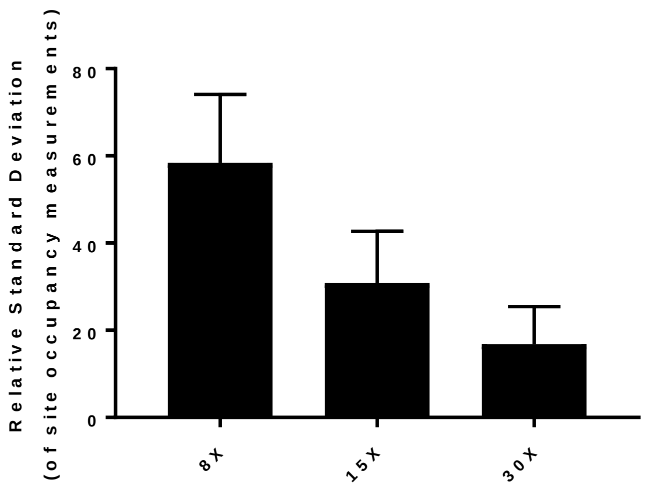

Supplementary Figure S2. Relative standard deviation of site occupancy measurments is smaller in samples with a higher DAR, likely due to higher signal values that are further from the limit of detection.

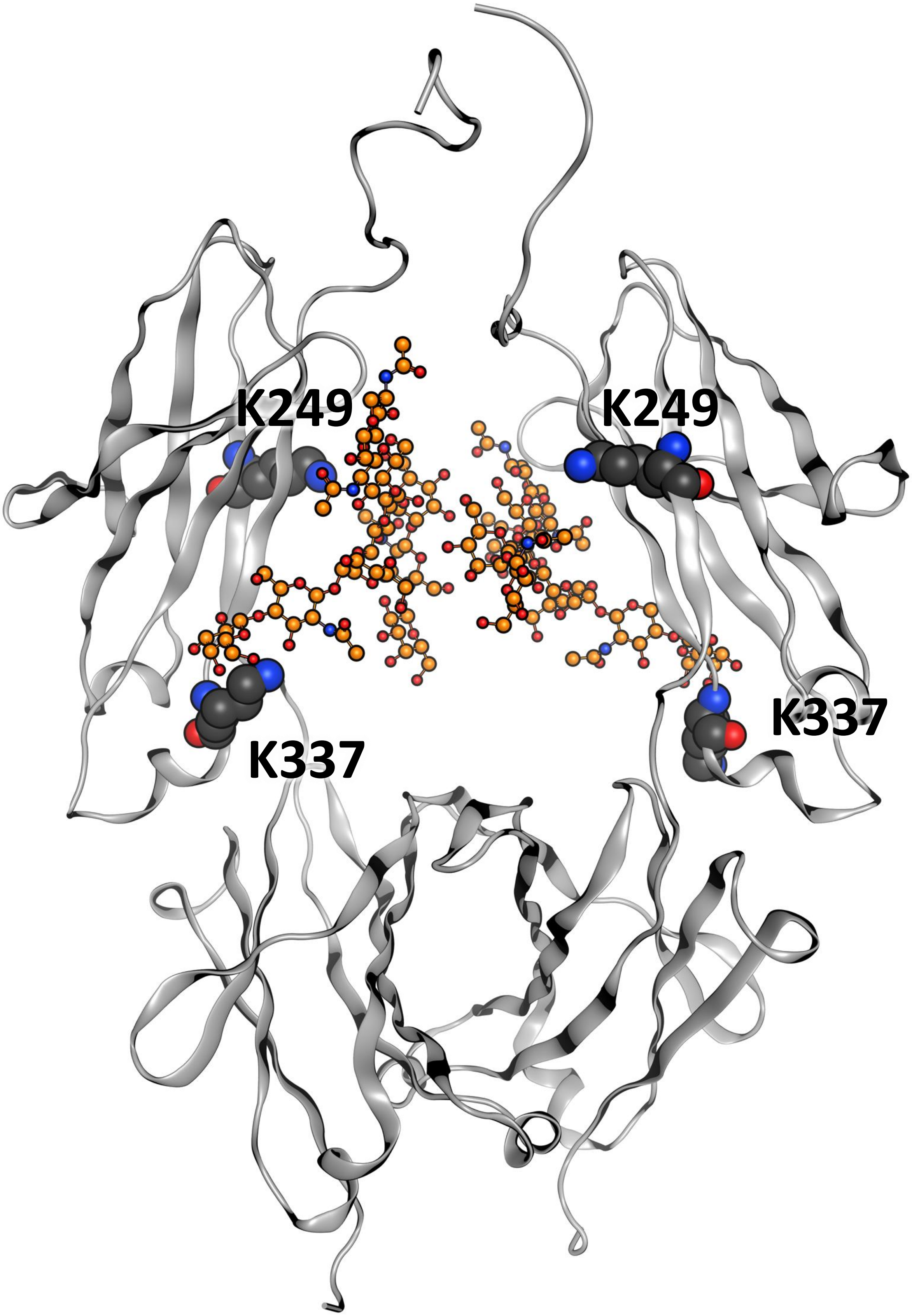

Supplementary Figure S3. Proximity of K249 and K337 (CPK models) to the carbohydrate structures (stick models) in the Fc region (PDB code 1HZH). The two protein chains of the Fc homodimer are shown in ribbon/tube rendering colored in green and in cyan.

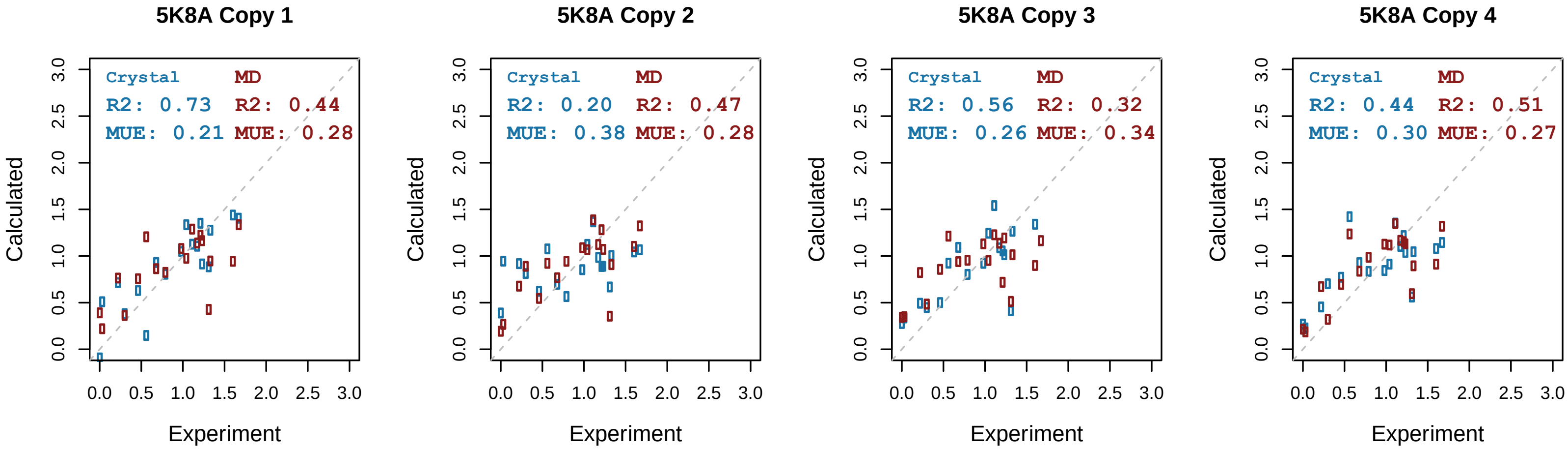

Supplementary Figure S4. Comparison of model training on single crystal conformation versus MD ensemble generated from each crystallographic conformation. Experimental single-lysine susceptibility data for the Fab fragment are from Table 1 in the main paper. See Methods for MD simulation details.

$$\text{Susceptibility} = 0.0160 * \text{SASA}_N - 0.3438 * \text{pKa}_{H++} + 4.1452$$

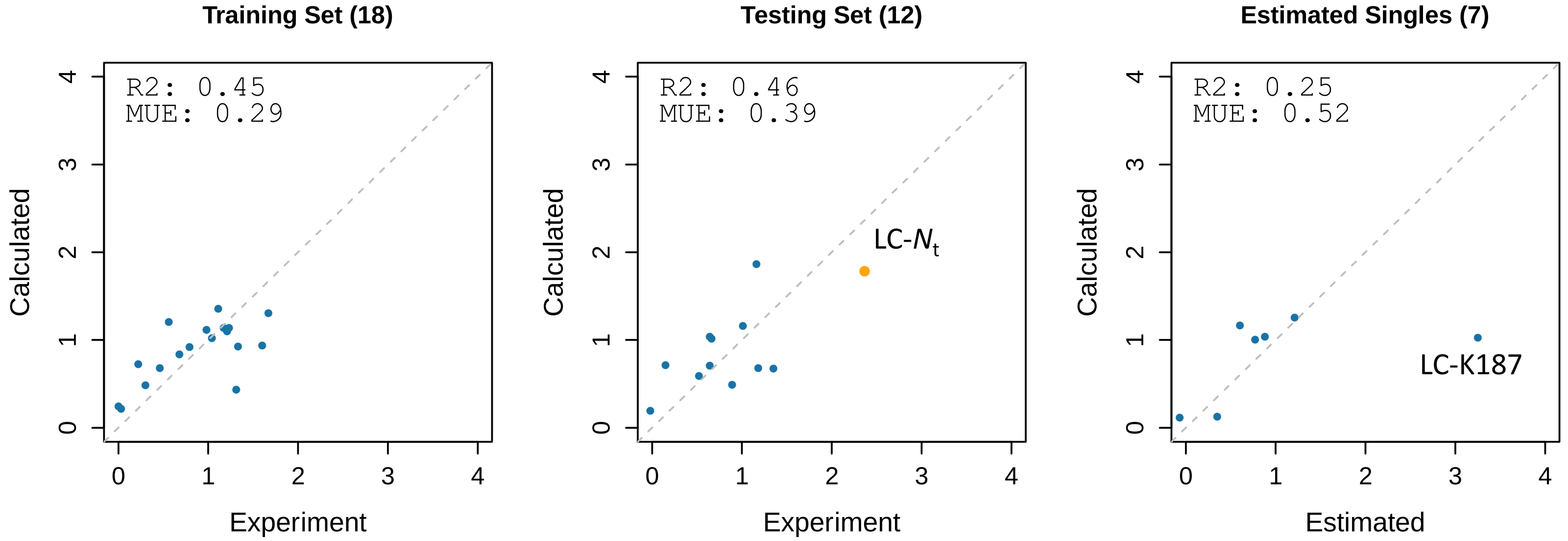

Supplementary Figure S5. Performance of a bi-parametric linear model trained on 4 combined MD ensembles for the Fab region of the NIST mAb. To be compared with the model in Eq (3) and Figure 7 from main paper which was derived on 4 crystal structure copies.

A.

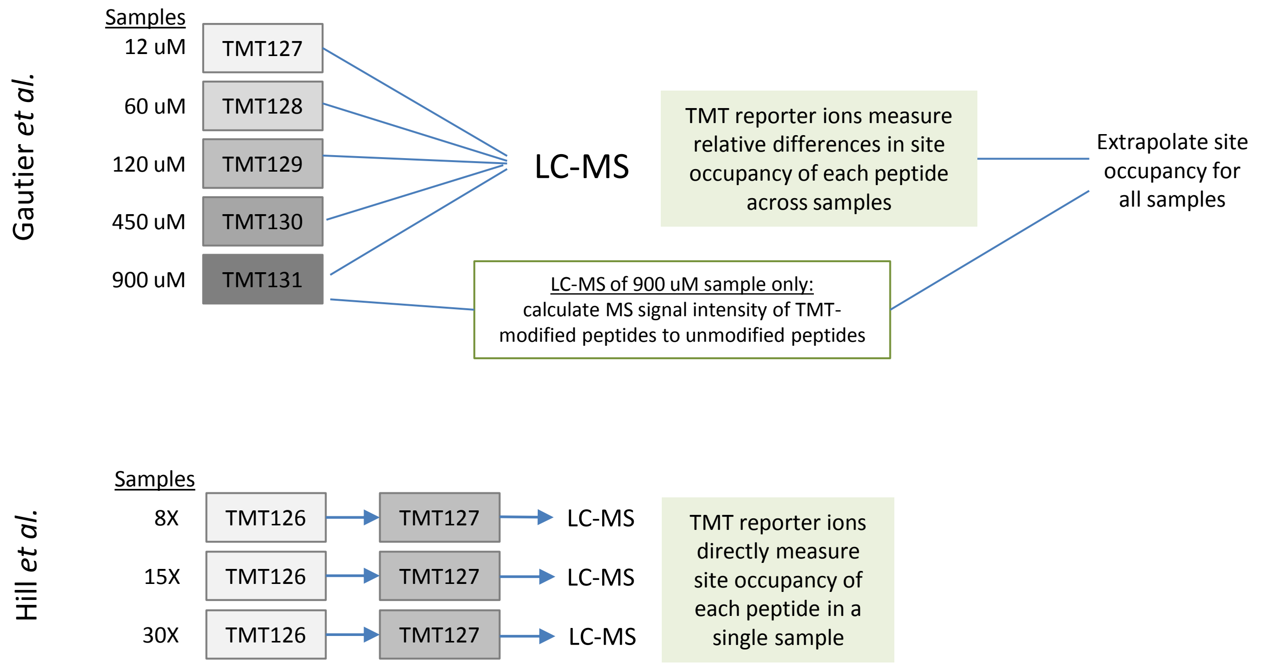

B.

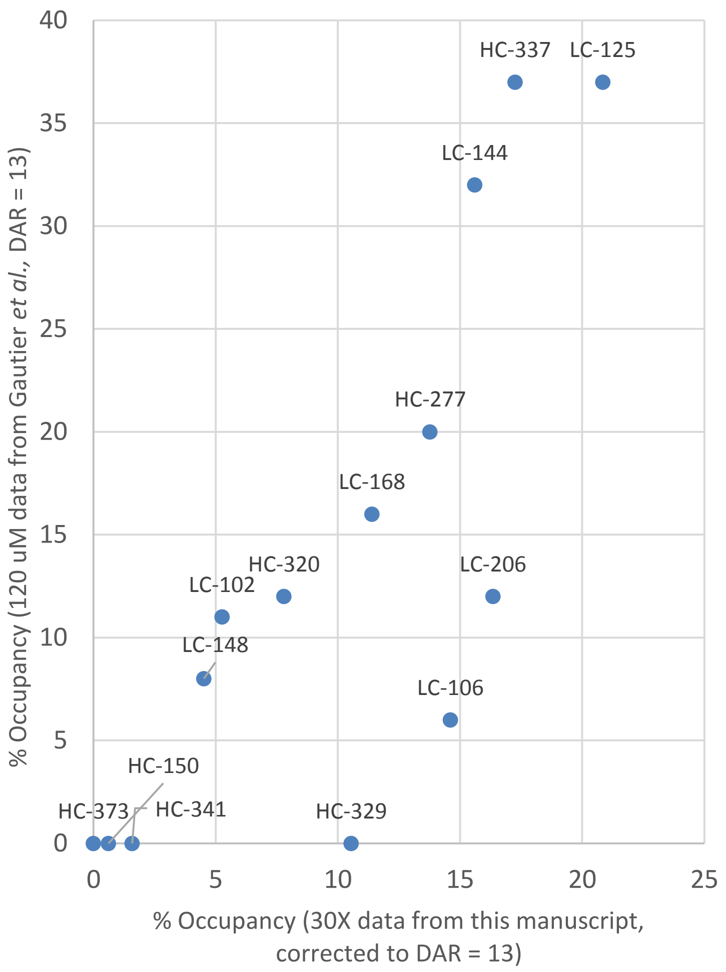

Supplementary Figure S6. (A) Comparison of the method described in this work (Hill *et al.*) with the method previously published by Gautier *et al.*[7]. (B) Comparison of the site occupancy values obtained for lysine residues present in the IgG1 constant regions that are common between this work and that previously published by Gautier *et al.*[7] Data points are labeled with the amino acid numbers from NIST mAb.
